# SUPPORT Tools for evidence-informed health Policymaking (STP) 10: Taking equity into consideration when assessing the findings of a systematic review

**DOI:** 10.1186/1478-4505-7-S1-S10

**Published:** 2009-12-16

**Authors:** Andrew D Oxman, John N Lavis, Simon Lewin, Atle Fretheim

**Affiliations:** 1 Norwegian Knowledge Centre for the Health Services, P.O. Box 7004, St. Olavs plass, N-0130 Oslo, Norway; 2 Centre for Health Economics and Policy Analysis, Department of Clinical Epidemiology and Biostatistics, and Department of Political Science, McMaster University, 1200 Main St. West, HSC-2D3, Hamilton, ON, Canada, L8N 3Z5; 3 Norwegian Knowledge Centre for the Health Services, P.O. Box 7004, St. Olavs plass, N-0130 Oslo, Norway; Health Systems Research Unit, Medical Research Council of South Africa; 4 Norwegian Knowledge Centre for the Health Services, P.O. Box 7004, St. Olavs plass, N-0130 Oslo Norway; Section for International Health, Institute of General Practice and Community Medicine, Faculty of Medicine, University of Oslo, Norway

## Abstract

*This article is part of a series written for people responsible for making decisions about health policies and programmes and for those who support these decision makers*.

In this article we address considerations of equity. Inequities can be defined as "differences in health which are not only unnecessary and avoidable but, in addition, are considered unfair and unjust". These have been well documented in relation to social and economic factors. Policies or programmes that are effective can improve the overall health of a population. However, the impact of such policies and programmes on inequities may vary: they may have no impact on inequities, they may reduce inequities, or they may exacerbate them, regardless of their overall effects on population health.

We suggest four questions that can be considered when using research evidence to inform considerations of the potential impact a policy or programme option is likely to have on disadvantaged groups, and on equity in a specific setting. These are: 1. Which groups or settings are likely to be disadvantaged in relation to the option being considered? 2. Are there plausible reasons for anticipating differences in the relative effectiveness of the option for disadvantaged groups or settings? 3. Are there likely to be different baseline conditions across groups or settings such that that the absolute effectiveness of the option would be different, and the problem more or less important, for disadvantaged groups or settings? 4. Are there important considerations that should be made when implementing the option in order to ensure that inequities are reduced, if possible, and that they are not increased?

## About STP

*This article is part of a series written for people responsible for making decisions about health policies and programmes and for those who support these decision makers. The series is intended to help such people ensure that their decisions are well-informed by the best available research evidence. The SUPPORT tools and the ways in which they can be used are described in more detail in the Introduction to this series *[1]. *A glossary for the entire series is attached to each article (see Additional File *1*). Links to Spanish, Portuguese, French and Chinese translations of this series can be found on the SUPPORT website *http://www.support-collaboration.org. *Feedback about how to improve the tools in this series is welcome and should be sent to: *STP@nokc.no.

## Scenario

*You work in the Ministry of Health. Improving drug insurance coverage for essential medicines is a government priority. The Minister of Health has asked you to present options for increasing coverage, including the expected impacts of such options on disadvantaged populations. You decide to commission a policy brief from a unit that supports the Ministry of Health in using evidence in policymaking. You ask them to pay particular attention to the likely impacts of alternative policies on inequities*.

## Background

**Figure 1:**
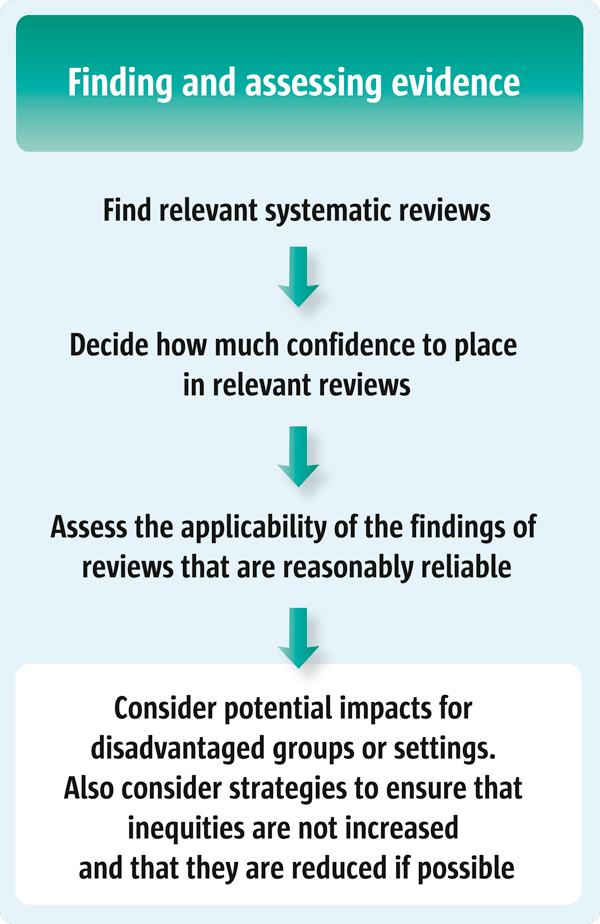
**Step 4 in finding and assessing systematic reviews to inform policymaking: equity considerations**.

In this article, which is the fourth in this series addressing the use of systematic reviews to inform policy decisions (see Figure 1), we suggest four questions that policymakers can consider when assessing the potential impacts a policy or programme is likely to have on disadvantaged populations and on equity. Such questions could be applied, for instance, in the scenario outlined above. For policymakers, such as a Health Minister or senior staff member in a Ministry, this article suggests a number of questions that staff might be asked to consider when preparing a policy brief regarding impacts on inequities. For those who support policymakers, such as those who are asked to prepare policy briefs, this article suggests questions that can be used to guide considerations when using research evidence regarding impacts on inequities, particularly when using evidence from systematic reviews [2].

We will not provide guidance for addressing inequities, which must be considered in relation to specific settings and policies. Rather, we will present a structured approach to considering the impacts of policy and programme options on inequities, to inform decisions about what options to implement and how to implement them.

Braveman and Gruskin define equity as "the absence of disparities in health that are systematically associated with social advantage or disadvantage" [3]. Margaret Whitehead emphasises the elements of disadvantage even more clearly by defining inequity as "differences in health which are not only unnecessary and avoidable but, in addition, are considered unfair and unjust" [4].

Inequities in health and healthcare are well documented in relation to a variety of social and economic characteristics. Disadvantaged populations almost always have poorer health [5], poorer access to healthcare [6], and receive poorer quality healthcare [7]. Policies or programmes that are effective can improve the overall health of the population. However, their impact on inequities may vary: they may have no impact on inequities, they may reduce inequities, or they may exacerbate them regardless of their overall effects on population health. It is therefore not sufficient for policymakers simply to know that a policy or programme is effective. They also need to consider how a policy or programme may impact on inequities. If it is likely to exacerbate these they also need to consider how such effects could be ameliorated. Many effective interventions to reduce smoking, for example, are taken up more readily by more advantaged groups, and this can lead to the widening of differences in smoking rates and health inequities if specific actions are not taken to address this.

## Questions to consider

The following questions can guide assessments of the potential impacts a policy or programme option is likely to have on disadvantaged populations and equity:

1. Which groups or settings are likely to be disadvantaged in relation to the option being considered?

2. Are there plausible reasons for anticipating differences in the relative effectiveness of the option for disadvantaged groups or settings?

3. Are there likely to be different baseline conditions across groups or settings such that that the absolute effectiveness of the option would be different, and the problem more or less important, for disadvantaged groups or settings?

4. Are there important considerations that should be made when implementing the option in order to ensure that inequities are reduced, if possible, and that they are not increased?

**Figure 2:**
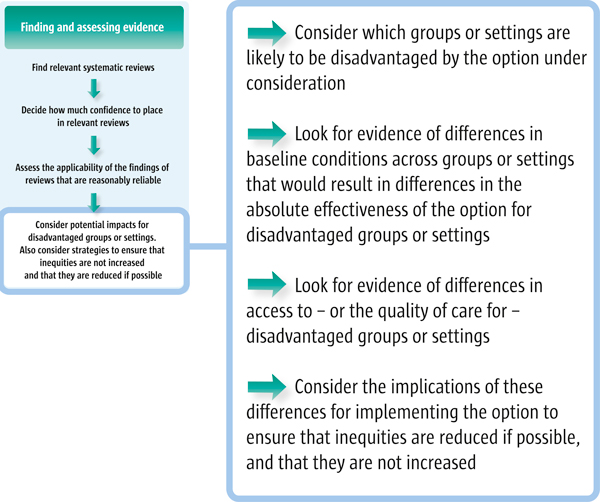
**Four steps to identifying and incorporating equity considerations when assessing the findings of a systematic review**.

The logic behind these questions is illustrated in Figure 2.

### 1. Which groups or settings are likely to be disadvantaged in relation to the option being considered?

Disadvantage may be related to economic status, employment or occupation, education, place of residence, gender, ethnicity, or combinations of these characteristics. Different societies give greater or lesser attention to particular factors due to historical circumstances. For example, in the United States there is often a greater focus on issues of race, while in the United Kingdom it is social class that draws attention. Other countries may focus on specific ethnic groups.

The relevance of these characteristics may vary depending on the policy or programme of interest. While there may be good reasons for prioritising particular groups or settings generally, for specific policies or programmes it is often important to consider inequities in relation to a range of potentially disadvantaged groups or settings. Subsequent attention should focus on those groups or settings for which there is a reason to anticipate significant differential effects.

Generally, researchers and policymakers should be concerned about differential effects whenever there is an association between the mechanism of action of the policy or programme, and particular characteristics. For example:

• *Economic status*: low-income populations are more likely to be responsive to changes in the prices of goods and services. Because they have less disposable income, tobacco tax increases, for example, could make such populations more likely to quit. But they would also be made more vulnerable as a result of having to spend more money on tobacco if they did *not *quit smoking

• *Employment or occupation*: employer-funded insurance schemes may result in differences in coverage, with less coverage being likely for those who are unemployed, self-employed or employed in small companies

• *Education*: school-based programmes would be expected to differentially affect those who attend versus those who do not attend schools. Information campaigns that rely on printed materials to improve the utilisation of health services might have differential impacts on illiterate or less-educated populations

• *Place of residence*: access to care is commonly more difficult in rural areas. Any strategy, therefore, that does not take into account the need to improve the delivery of effective clinical or public health interventions is likely to be less effective in rural areas

• *Gender*: strategies for involving stakeholders in priority-setting may affect women and men differently, resulting in priorities that may have different impacts on women and men

• *Ethnicity*: ethnic groups (e.g. those groups who consider themselves, or are considered by others, to share common characteristics which differentiate them from other groups in society [8]) may have beliefs and attitudes relating to the acceptability of a particular policy or programme. Delivery strategies that do not take these perspectives into account are likely to be less effective amongst ethnic groups where an otherwise effective policy or programme might not be readily accepted

### 2. Are there plausible reasons for anticipating differences in the relative effectiveness of the option for disadvantaged groups or settings?

In Table 1 we present an example of a scenario in which one might anticipate differences in the relative effectiveness of a policy or programme. As described in the Table, there are plausible reasons for anticipating differences in the relative effects of requiring user fees to pay for drugs or other health services on disadvantaged populations (such as the poor), compared to other populations that are not disadvantaged. When attempting to reduce disparities in such circumstances, policymakers should look for evidence of the impacts of the considered options on relevant disadvantaged populations. This evidence should be taken into consideration when deciding what action to take. For example, should user fees be used at all? And if they are used, how could they be designed and implemented in order to minimise their adverse effects on the poor?

**Table 1:** An example of a plausible reason for anticipating differences in relative effectiveness

User fees were widely introduced in sub-Saharan Africa as part of the Bamako Initiative adopted by Health Ministers of the WHO African Region in 1988 [27]. The Initiative advocated selling drugs to users at a profit: the intention was to use the profit, in addition to user consultations payments, to improve access to care and quality of service. Opinion remains divided on the impact of introducing user fees for accessibility to services, particularly on the very poor. This initiative has been the subject of much debate for more than 15 years but there can be no doubt that user fees are a financial barrier for poor people needing drugs or other health services [28,29].
In other instances where a third party pays all drug costs, patients may potentially have inappropriately high utilisation rates [30]. Direct cost-share policies shift part of the financial burden from insurers to patients and therefore increase patient financial responsibility for prescription drugs. These policies are intended to be an incentive to reduce the following: the overall overuse of drugs; the use of drugs of limited efficacy or those used for conditions where other, more cost-effective treatments are available; and third party payer expenditures. Patients are expected to respond to direct payments by decreasing drug use, by shifting to cheaper drugs, or by paying more costs out-of-pocket. By reducing the financial burden for third party payers and facilitating rational drug use, overall health levels may be improved by saving resources and reallocating them to other healthcare services.
However, a too-restrictive drug insurance policy may have unintended consequences. For example, a shift of cost from insurer to consumer may lead to the discontinuation of necessary drugs by patients. In turn, this may cause a deterioration of health and an increase in healthcare utilisation and expenditures for both patients and insurers. This is an unintended effect that is likely to have a larger impact amongst low-income or other vulnerable populations because such costs are likely to represent a more substantial proportion of total income. Schemes involving direct payment for drugs by patients are therefore controversial because increased cost sharing for drugs may present a financial barrier to the poor and other disadvantaged groups. Placing a cap on reimbursement for prescriptions has been shown to be linked to a reduction in the use of essential drugs in vulnerable subgroups of both elderly patients and severely disabled patients, and increases in hospitalisations and nursing home admissions [30].

Evidence of the effects of policies or programmes on inequities is sparse. Finding this evidence is also difficult [9], and publication bias may be an additional problem given that studies identifying statistically significant differences in effects are more likely to be published than those that do not [9]. Tsikata and colleagues, for instance, found that only 10% of controlled trials assessed the efficacy of a policy or programme across socio-economic subgroups [10]. Similarly, Ogilvie and colleagues found that Cochrane reviews of studies of tobacco control rarely assessed the impact of the policy or programme across socio-economic factors, both in the actual reviews and the primary studies in those reviews [11]. Systematic reviews generally tend not to provide evidence of differential effectiveness [11-15]. Because of this, it may be necessary to search for a wider scope of evidence than that which is typically found in systematic reviews. Such evidence may be needed to support or refute plausible hypotheses of differential effects, or the effects of policies or programmes on reducing inequities.

When subgroup analyses are undertaken in systematic reviews to explore whether there are differential effects, policymakers should be aware that these can be misleading. This is because studies may be too small to reliably detect differences in effects, resulting in false negative conclusions. Also, testing multiple hypotheses regarding factors that might moderate the effectiveness of a policy may result in false positive conclusions [16-20]. The results observed in subgroups, for instance, may differ by chance from the overall effect observed across studies [18,21]. Paradoxically, the best estimate of the outcome of a policy or programme in a subgroup may be the *overall *results (across different subgroups) rather than the specific results for the subgroup of interest [18,22,23]. General guidelines for interpreting subgroup analyses (see Table 2) should be applied with a healthy scepticism whenever subgroup analyses, including subgroup analyses based on socio-economic factors, are considered [24].

**Table 2:** Guidelines for interpreting subgroup analyses

The following questions can help in the process of deciding whether a decision should be based on a subgroup analysis or the overall results:
**Is the magnitude of the difference important?**
If the magnitude of a difference between subgroups will not result in different decisions for different subgroups, then the overall results can be used.
**Is the difference between subgroups statistically significant?**
To establish whether a policy or programme has a different effect in different situations, the magnitudes of effects in different subgroups should be compared directly with each other. The statistical significance of the results within separate subgroup analyses should *not *be compared, as this is likely to be misleading. For example, if a subgroup analysis showed that the effect of a policy or programme was not statistically significant for women but was statistically significant for men, it is likely that this could simply be because few women were included in the studies. It does *not *answer the question of whether the difference between the size of the effect in women and men was greater than would otherwise have been expected if this had occurred by chance. If there is both an important difference in effects and that difference is statistically significant (i.e. it is unlikely to have occurred by chance), then serious consideration should be given to basing a decision on the subgroup analysis rather than on the overall analysis.
**Is there indirect evidence in support of the findings?**
Indirect evidence is research that has not directly compared the options in which we are interested in the populations in which we are interested, or measured the important outcomes in which we are interested. For differences between subgroups to be convincing, they should be plausible and supported by other external or indirect evidence. For example, research that has measured intermediary outcomes (not the ones in which we are interested) can provide evidence of a plausible mechanism for differential effects. For subgroup analyses for disadvantaged groups, there should be a similarly plausible reason - supported by indirect evidence - to anticipate differential effects.
**Was the analysis pre-specified or post hoc?**
Researchers should state whether subgroup analyses were pre-specified or undertaken after the results of the studies had been compiled (post hoc). Greater reliance may be placed on a subgroup analysis if it formed part of a small number of pre-specified analyses. Performing numerous post hoc subgroup analyses could be seen as data dredging, a process that is inherently unreliable. This is because it is usually possible to find an apparent - but false - explanation for differences in effects when considering many different characteristics.
**Are analyses looking at within-study or between-study relationships?**
Differences in subgroups that are observed within studies are more reliable than analyses of subsets of studies. If such within-study relationships are replicated across studies then this will add confidence to the findings.

Similarly, there is often a lack of direct evidence related to disadvantaged populations given that they may not actually have been included in studies. In these circumstances, policymakers need to consider the applicability of the available evidence, as discussed in Article 9 in this series [25].

### 3. Are there likely to be different baseline conditions across groups or settings such that that the absolute effectiveness of the option would be different, and the problem more or less important, for disadvantaged groups or settings?

**Figure 3:**
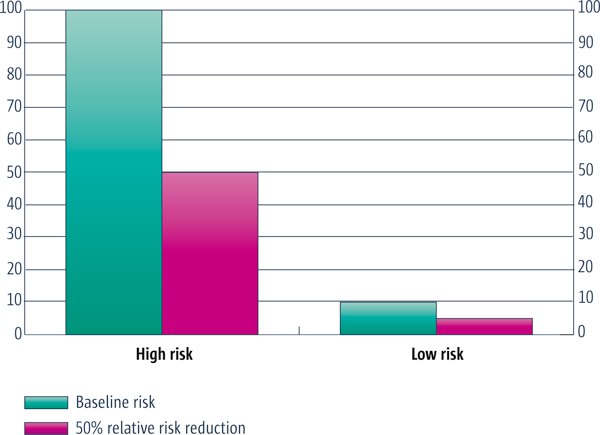
**Absolute versus relative reductions in risk**.

If the *relative *effectiveness of a policy or programme is similar in disadvantaged settings, there may still be important differences in the *absolute *effect due to differences in baseline conditions (see Figure 3 for an illustration, Table 3 for an example, and Table 4 for an explanation of relative and absolute effects). Typically, baseline risks are larger in disadvantaged populations and a larger absolute effect could therefore be expected. If the relative effect of improving the delivery of artemisinin combination therapy (ACT) on mortality from malaria is the same for disadvantaged children as it is for other children, for example, the absolute effect would be greater in disadvantaged populations that have a higher mortality rate. Risks may occasionally be lower in disadvantaged populations and, in these instances, the absolute effect will also consequently be less. The baseline risk for coronary artery disease among Filipinos is about one-fifth of the baseline risk in the United States. Therefore the number of people it is necessary to treat (and the corresponding cost) in order to prevent one case of coronary artery disease, is five times greater among Filipinos.

**Table 3:** An example of a difference in baseline conditions leading to a difference in absolute effectiveness

Facility-based births can help to reduce maternal mortality when such facilities are appropriately equipped and staffed by skilled health workers who are able to deliver effective interventions to reduce deaths from the common causes of maternal deaths such as haemorrhage and eclampsia. Typically proportions of facility-based births are lower in rural areas than in urban areas due to variations in accessibility. Paying transportation costs to improve access to facilities might reduce inequities. This is because payments may be more effective in rural areas where transportation costs are more of a barrier. It is also due to the lower proportion of facility-based births in rural areas (which thus increases the absolute effect).

**Table 4:** Relative and absolute effects

**Relative effects **are ratios. For example, a risk ratio (RR) is the ratio between the risk in an intervention group and the risk in a control group. If the risk in an intervention group is 2% (i.e. 20 per 1,000) and the risk in a control group is 2.4% (i.e. 24 per 1,000), the risk ratio (or relative risk) will be 20/24 or 83%. 'Relative risk reduction' is another way of expressing relative effects. This is the proportional or percentage reduction in risk, and is equal to 1-RR which, in this case, is 17% (1 - 0.83 = 0.17).
If the RR value is exactly 1.0, this means that there is no difference between the occurrence of the outcome in the intervention group and the control group. But the significance of this value being above or below 1.0 depends on whether the outcome being measured is judged to be good or bad. If the RR value is greater than 1.0, the intervention increases the risk of the outcome. If the desired outcome is considered to be good (for example, the birth of a healthy baby), an RR greater than 1.0 indicates a desirable effect for the intervention. Conversely, if the outcome is bad (for example, death) an RR value greater than 1.0 would indicate an undesirable effect. If the RR value is less than 1.0, the intervention decreases the risk of the outcome. This then indicates a desirable effect, if it is a bad outcome (for example, death) and an undesirable effect if it is a good outcome (for example, the birth of a healthy baby).
**Absolute effects **are differences. For example, absolute risk reduction (ARR) is the difference between the risk *with *the intervention and the risk *without *the intervention. In this example, the ARR is 2.0% (20 per 1,000) minus 2.4% (24 per 1,000) i.e. 0.4% (4 per 1,000) fewer deaths from bowel cancer.
Usually the absolute effect is different for high-risk groups (such as those who are disadvantaged) and low-risk groups, whereas the relative effect is often the same. When relevant, it is therefore important to consider whether different groups have different levels of risk. This is illustrated in Figure 3, where a 50% relative reduction in risk is shown to result in an absolute reduction of 50 events per 1,000 in the high risk group (from 100 to 50) and an absolute reduction of only 5 per 1,000 in the low risk group (from 10 to 5).

### 4. Are there important considerations that should be made when implementing the option in order to ensure that inequities are reduced, if possible, and that they are not increased?

Disadvantaged populations generally have poorer access to care and often receive poorer quality care. This is particularly true for hard-to-reach populations, such as illegal immigrants. Consequently, programmes to improve access and the quality of care will often require implementation strategies tailored to address factors that limit access or quality in disadvantaged settings or groups (see Table 5, for example). Such methods may include different delivery, financial and governance strategies, or the investment of additional resources. They may also include the provision of additional technical support to implement non-tailored strategies for such groups.

**Table 5:** An example of important considerations regarding implementation

There is a greater likelihood that disadvantaged children compared to more advantaged children will be exposed to greater health risks, have less resistance to disease, and will therefore have higher mortality rates. These inequities are compounded by reduced access to health services. Even public subsidies for health frequently benefit rich people more than poor people. Implementing interventions to reduce child mortality will not necessarily reduce these inequities and may, in some cases, even increase them. Consideration should thus be given to strategies designed to reduce inequities, such as the provision of more affordable and accessible health services [31]. These strategies may target poor people or they may be implemented universally. Situations in which targeting or universal coverage might be more appropriate include [31]:	
	

**Targeting more likely to be appropriate**	**Universal coverage more likely to be appropriate**

• High risk groups easy to identify	• High risk groups hard to identify
• Intervention only needed by children at risk	• Intervention needed by everyone
• Intervention only protects those who receive it	• Intervention has a spill-over effect
• Intervention is widely provided through the public sector	• Intervention is widely provided through the private sector
• Spontaneous demand for the intervention is low	• Spontaneous demand for the intervention is high
• Health services are unable to cover the whole population	• Health services are able to cover the whole population

	

Universal coverage may be a more appropriate strategy for vaccines, which are needed by everyone and which have spill-over effects (decreasing the risk of infection for both those who are vaccinated and others). However, in order to also reduce inequities in coverage, additional targeted strategies may be needed such as those that address problems with regard to differences in health service accessibility or to a lack of demand for vaccinations in disadvantaged populations.	

## Conclusion

Policymakers can expect to find limited evidence of the impacts of most health policies on inequities. When they are presented with subgroup analyses that explore whether there are different impacts on specific disadvantaged groups or settings, they should recognise that these analyses may be misleading. Many policies or programmes may, in fact, have similar relative effects in disadvantaged settings and elsewhere. Nonetheless, differences in absolute effects (due to differences in baseline risks or needs) and differences in barriers to implementing them, are likely to be common. The evidence for such differences should be considered and taken into account when making policy decisions. Because the evidence is often limited, it is important to ensure that the monitoring and evaluations of impacts on equity are as rigorous as possible to ensure that intended effects are achieved and that unintended adverse effects are avoided.

To monitor or evaluate the extent to which implementing policies or programmes differentially affects disadvantaged populations, policymakers should ensure that appropriate indicators of social gradients and measures of change are used. When the reduction of inequities is a priority for policymakers, they should look beyond considerations related to the impacts of health system arrangements on disadvantaged populations. They may also want to consider potential strategies for addressing the social determinants of health and the evidence supporting those strategies [26].

## Resources

### Useful documents and further reading

Improving the use of research evidence in guideline development: 2. Incorporating considerations of equity. Health Res Policy Syst 2006; 4:24.

- http://www.health-policy-systems.com/content/4/1/12 - This article reviews the literature on incorporating considerations of equity in guidelines and recommendations

- Dans AM, Dans L, Oxman AD, Robinson V, Acuin J, Tugwell P, Dennis R, Kang D. Assessing equity in clinical practice guidelines. J Clin Epidemiol. 2007; 60:540-6. http://www.ncbi.nlm.nih.gov/pubmed/17493507 - This article discusses criteria for users to evaluate how well clinical practice guidelines address issues of equity

- Braveman PA and Gruskin S. Defining equity in health. J Epidemiol Community Health 2003; 57:254-8. http://jech.bmj.com/cgi/content/full/57/4/254

- Whitehead M. The concepts and principles of equity and health. Int J Health Serv 1992; 22:429-45. http://www.ncbi.nlm.nih.gov/pubmed/1644507

- Tugwell P, de Savigny D, Hawker G, Robinson V. Applying clinical epidemiological methods to health equity: the equity effectiveness loop. BMJ 2006; 332:358-61. http://www.bmj.com/cgi/content/full/332/7537/358

### Links to websites

Although the focus of this article (and others in this series) is on policies within the health sector, we have included links to websites that also focus more broadly on the determinants of health. These are relevant to evidence-informed policymaking both within and outside the health sector.

- Archives of equidad@listserv.paho.org - This is the archive of the Pan American Health Organization's (PAHO's) EQUIDAD list. Messages sent to the list cover a broad range of material, both in published and grey literature, and address all aspects of equity in health as well as other health systems topics.

- Cochrane Health Equity Field: http://equity.cochrane.org/en/index.html - The Cochrane Health Equity Field forms part of the Cochrane Collaboration http://www.cochrane.org. It is co-registered with the Campbell Collaboration http://www.campbellcollaboration.org as the Campbell Equity Methods Group. This Field encourages and supports the authors of systematic reviews to include explicit descriptions of the effects of interventions on the disadvantaged and the ability of interventions to reduce inequalities.

- European Portal for Action on Health Equity: http://www.health-inequalities.eu - This portal is a tool to promote health equity amongst different socio-economic groups in the European Union. It provides information on policies and interventions to promote health equity within and between the countries of Europe.

- WHO - Commission on Social Determinants of Health: http://www.who.int/social_determinants/en - The final reports on the WHO Commission on Social Determinants of Health are available here. They are intended to support countries and global health partners to address the social factors leading to ill health and inequities. These reports draw attention to the social determinants of health that are known to be among the worst causes of poor health and inequalities between and within countries. The determinants include unemployment, unsafe workplaces, urban slums, globalisation and a lack of access to health systems.

- World Bank - Multi-Country Projects in Equity, Poverty, and Health: http://web.worldbank.org/WBSITE/EXTERNAL/TOPICS/EXTHEALTHNUTRITIONANDPOPULATION/EXTPAH/0,,contentMDK:20219025~menuPK:460198~pagePK:148956~piPK:216618~theSitePK:400476~isCURL:Y,00.html - Recent increases in concern related to the health of the poor have given rise to a large number of inter-country research projects on poverty, equity and health. This website provides links to other resources for information on equity, poverty and health.

- EQUINET Africa: http://www.equinetafrica.org - EQUINET, the Regional Network on Equity in Health in Southern Africa, is a network of professionals, civil society members, policymakers, state officials and others within the region who have come together as an equity catalyst, to promote and realise shared values of equity and social justice in health.

- Global Equity Gauge Alliance: http://www.gega.org.za - The Global Equity Gauge Alliance was created to support an active approach to monitoring health inequalities and to promote equity within and between societies. The Alliance currently includes 11 member-teams, called Equity Gauges, located in 10 countries in the Americas, Africa and Asia.

## Competing interests

The authors declare that they have no competing interests.

## Authors' contributions

ADO prepared the first draft of this article. JNL, SL and AF contributed to drafting and revising it.

## Supplementary Material

Additional file 1 Glossary Click here for file
